# The mediating effect of mindfulness on adverse childhood experiences and psychological resilience in Turkish female university students

**DOI:** 10.3389/fpsyg.2025.1528159

**Published:** 2025-08-08

**Authors:** Elif Sezgin

**Affiliations:** Healthy Science Faculty, Child Development, Mudanya University, Bursa, Türkiye

**Keywords:** mindfulness, psychological resilience, adolescent, childhood trauma, adverse childhood experience

## Abstract

**Introduction:**

Adverse childhood experiences (ACEs) and childhood traumas are known to have lasting negative effects on psychological wellbeing. These early adverse experiences can significantly impair psychological resilience and the capacity to adapt to adversity. While mindfulness has been consistently associated with higher resilience, its potential buffering or mediating role in the relationship between early traumatic experiences and resilience remains underexplored. Although many studies have examined ACEs, fewer have distinguished between general adverse experiences and more severe childhood traumas. This study aimed to determine whether mindfulness mediates the relationship between ACEs, childhood traumas, and resilience among female university students in Turkey. The mindfulness scale used in the study was designed to assess mindfulness and covers four main dimensions: attention and awareness, non-reactivity, non-judgment, and self-acceptance. The possible mediating effect of mindfulness through these dimensions was analyzed.

**Methods:**

Data were obtained through self-reporting from 255 female university students enrolled in a vocational school in Bursa, Turkey. The survey collected information on ACEs and childhood traumas (emotional abuse, physical neglect, etc.), mindfulness levels (with a focus on attention and awareness), and resilience. Statistical analyses, including mediation analysis, were performed to assess relationships between these variables.

**Results:**

A negative correlation was found between ACEs, including specific forms such as emotional abuse and physical neglect, and resilience. Among these, physical neglect showed the strongest negative association with mindfulness, particularly in the attention and awareness dimension. Furthermore, this dimension of mindfulness significantly predicted higher levels of psychological resilience. Mediation analysis revealed that mindfulness partially mediated the relationship between ACEs, childhood traumas, and resilience. These findings suggest that individuals with higher mindfulness, especially in attention and awareness, may be better protected against the long-term negative impacts of early adverse experiences and trauma on resilience.

**Discussion:**

The findings highlight that emotional abuse and physical neglect significantly reduce mindfulness levels in female university students, with physical neglect having the most substantial impact on attention and awareness. Since mindfulness, particularly attentional awareness, positively predicts resilience, interventions enhancing mindfulness may help mitigate the long-term effects of ACEs. Future research should explore these relationships in broader populations and longitudinal designs.

## Introduction

Adverse childhood experiences (ACEs) refer to potentially stressful events or environments that occur before the age of 18. These include various types of abuse—such as physical, emotional, and sexual abuse—as well as forms of neglect, including both physical and emotional neglect. ACEs are also assessed in the context of household dysfunction, such as parental separation, incarceration of family members, and mental illness within the household (Senaratne et al., 2024).

Prevalence studies indicate that a significant proportion of adolescents are exposed to at least one potentially traumatic event before the age of 18. For instance, Pérez et al. (2017) found that 77% of 422 adolescents aged 10–19 reported experiencing a traumatic event. Similarly, Astitene et al. (2018) reported that 88.69% of 871 adolescents had encountered at least one traumatic experience in their lifetime. Suliman et al. (2005) also found that 91% of 11th-grade students in their study had experienced trauma.

ACEs represent a significant global public health concern due to their long-term impact on children and adolescents. Research has shown that exposure to such experiences contributes to the development of psychological and behavioral problems throughout adolescence and young adulthood (Mao et al., 2022; Li et al., 2022). These include increased risks for post-traumatic stress, anxiety, and depression (Pérez et al., 2017). Rollocks et al. (2013) found that adolescents with multiple traumatic experiences reported higher levels of anxiety, anger, depression, and PTSD symptoms.

In a study of university students, Aydin et al. (2009) identified separation from a caregiver (46.1%), witnessing violence (33.1%), harsh punishment (21.2%), and family substance use (10.5%) as common childhood traumas. The findings indicated that these experiences were associated with higher levels of dissociation and general psychopathology. Similarly, Dobos et al. (2021) reported that female university students scored higher in difficulties with emotion regulation, anxiety, and perceived lack of social support compared to males, with difficulties in emotion regulation positively linked to childhood trauma. Kaloeti et al. (2019) also demonstrated a direct relationship between childhood trauma and depressive symptoms, while Li et al. (2022) found that childhood trauma correlated positively with aggression and negatively with psychological resilience. These studies collectively emphasize the long-term psychological effects of childhood trauma, including dissociation, depression, anxiety, aggression, and emotional regulation problems. Nevertheless, outcomes may vary depending on individual coping mechanisms and levels of resilience (Mao et al., 2022).

The adverse effects of trauma can be mitigated through the development of mindfulness, particularly in adolescents who have experienced ACEs. Mindfulness refers to a state of intentional awareness, where individuals focus on the present moment in an open and non-judgmental manner (Kabat-Zinn, 2005; Kaunhoven and Dorjee, 2017). Research suggests that mindfulness can serve a protective function for adolescents exposed to ACEs (Huang C.-C., 2021; Kachadourian et al., 2021) and that interventions targeting mindfulness skills may positively influence psychological wellbeing (Cutright et al., 2019).

### ACEs, mindfulness, and psychological resilience

The relationship between psychological resilience, ACEs, childhood trauma, and mental health outcomes is complex and multidimensional. Psychological resilience is defined as an individual's capacity to adapt positively and recover from adversity, trauma, or significant stress (Peng et al., 2012; Elisei et al., 2013). Numerous studies have shown that resilience helps reduce the negative impact of early adversities on mental health and promotes psychological wellbeing (Horton et al., 2022; Broche-Pérez and Jiménez-Morales, 2024).

ACEs are known to disrupt healthy psychological development and increase vulnerability to mental health issues later in life. However, resilience can serve as a buffer against these harmful outcomes, supporting recovery and reducing the risk of secondary traumatic stress (Özbay and Bülbül, 2025; Dagli and Topkara, 2023; Dhungana et al., 2022; Ungar and Theron, 2020). For example, Hu et al. (2024) found that resilience plays an important role in the relationship between childhood trauma and psychotic-like experiences (PLEs). The findings suggest the potential benefits of clinical practices designed to enhance resilience in the prevention and intervention of PLEs among university students.

Among strategies aimed at enhancing resilience, the development of mindfulness skills has garnered increasing attention. Lately, mindfulness has attracted considerable interest in psychological research. It refers to the deliberate and accepting awareness of one's present experiences, such as thoughts, feelings, and bodily sensations, without judgment (Kabat-Zinn, 2003). This mindful awareness enables people to step away from worries about the past or future and engage with their current experiences in a more open and accepting manner. Kabat-Zinn (2003) emphasized mindfulness primarily in the contexts of stress reduction and wellbeing. Literature views mindfulness as both a temporary mental state and a trainable skill linked to cognitive functions such as emotion regulation, attention, and self-awareness (Baer, 2003; Bishop et al., 2004; Gülden and Yalçin, 2024). Jiang et al. (2023) demonstrated that mindfulness fosters a more positive cognitive framework, enhancing resilience among university students with depressive symptoms during stressful periods. Higher levels of mindfulness are associated with greater resilience, while lower levels are linked to decreased coping capacity (Liu et al., 2022). This positive relationship has been confirmed across various cultural and demographic groups, including adolescents and university students (Zhang et al., 2024; Zuo et al., 2023; Chen et al., 2021a). Moreover, several studies have shown that mindfulness-based practices enhance resilience and reduce emotional and behavioral difficulties (Huang et al., 2019; Aini et al., 2021).

One possible explanation for the positive relationship between mindfulness and resilience is as follows: First, mindfulness assists individuals accept ongoing internal and external experiences without judgment. This encourages resilience and fosters a more accepting approach to life experiences (Vo et al., 2024). Individuals with high levels of mindfulness typically adopt a problem-solving approach, as they tend to engage in less rumination and worry. Those with heightened mindfulness are better equipped to manage their thoughts and emotions without feeling overwhelmed or stressed. As a result, they exhibit greater resilience (Liu et al., 2022; Mancini, 2021).

In recent years, researchers have begun to examine the relationship between mindfulness and psychological resilience, focusing on the effects of these factors on mental health. However, it is emphasized that more comprehensive and in-depth research is needed on the nature and strength of the relationship between these two concepts (Mancini, 2021). Significant evidence in the literature supports the relationship between ACEs, resilience, and mindfulness, but most research has been conducted in Western countries. Therefore, the generalizability of these findings to individuals living in non-Western countries and cultures is uncertain (Huang C.-C., 2021). Studies do not provide a clear consensus about the differences in adverse experiences and their consequences across these cultural domains, suggesting that more research is needed to clearly define these distinctions (Jobson et al., 2022, 2024). On the other hand, most ACEs studies focus on psychological dysfunction, and less is known about how ACEs are associated with other positive psychological outcomes, including resilience (Chen et al., 2023).

Investigating gender-specific aspects of resilience during young adulthood may reveal culturally relevant aspects of *psychological* resilience. It is important to note that the application of psychological resilience to broader populations of young women has thus far been under-researched and identified as a research gap (Haffejee and Theron, 2019; Jefferis and Theron, 2018). Findings from this study support other related research by contributing to gender-specific resilience mechanisms.

Additionally, there are a number of studies on the psychological resilience of female university students, especially abroad (see McKnight and Loper, 2002; Kirmani et al., 2015; Andersen et al., 2011; Satyanarayana et al., 2016). However, these studies have generally focused on early and middle adolescence. Research specifically on psychological resilience in female university students is limited, to the best of my knowledge (e.g., Kajbafnezhad and Khaneh Keshi, 2015; Athanasiades et al., 2023; Johnson et al., 2022; Maniram, 2022; Bhave et al., 2024). Generally, comparisons of resilience between male and female university students have been examined (e.g., Katyal, 2014; Allan et al., 2014; Li H. et al., 2023; Sojer et al., 2024; Vera Gil, 2024). In Turkey, no research has been conducted specifically on the psychological resilience of female university students. Therefore, this study is intended to shed light on gender-related cultural differences in psychological resilience.

On the other hand, very few studies in the literature examine the relationship between mindfulness and psychological resilience specifically in female university students (e.g., Kirmani et al., 2015; Anastasiades et al., 2017). A study investigating the link between psychological resilience and mindfulness levels in this population represents an important scientific field. Women between the ages of 18 and 21 face various challenges, such as academic stress, social integration, and emotional development. At this stage, psychological resilience and mindfulness are pivotal in coping with these challenges and improving overall mental health. Examining the link between mindfulness and psychological resilience, especially in female university students who have experienced ACEs, may provide valuable insights for creating practical intervention approaches. Investigating the mindfulness levels and psychological resilience of female university students—especially those who have experienced ACEs—can offer important guidance for developing future intervention strategies (Dawson et al., 2020; Galante et al., 2018).

There is a critical need to investigate whether ACEs experienced by female university students in Turkish society affect both their psychological resilience and mindfulness, as well as the relationship between mindfulness and resilience, similar to findings in Western and other countries. Due to the lack of research on the role of mindfulness in the psychological resilience of female university students experiencing ACEs in non-Western countries, and the importance of university years in determining adult wellbeing (Costa et al., 2013; Marginson, 2018), this study aims to examine the effects of ACEs on psychological resilience and mindfulness levels and to determine the role of mindfulness in the relationship between ACEs and psychological resilience. It also aims to investigate the relationship between ACEs, psychological resilience, and mindfulness. Studies in the literature on the effects of ACEs on the psychological resilience and mindfulness levels of female university students specifically are limited. This study aims to fill the knowledge gap by examining the relationship between ACEs, mindfulness, and psychological resilience. For this study, the following hypotheses focus on the relationships among ACEs, psychological resilience, and mindfulness, as well as the mediating role of mindfulness:

This study tested three interrelated hypotheses concerning the associations among ACEs, childhood trauma, mindfulness, and psychological resilience in female university students. It was hypothesized that higher levels of ACEs and Childhood Trauma Questionnaire (CTQ) scores would be significantly associated with lower levels of psychological resilience. Furthermore, it was expected that ACEs and CTQ scores would also be negatively associated with mindfulness levels. Finally, it was hypothesized that mindfulness would mediate the relationship between ACEs, CTQ scores, and psychological resilience, suggesting that higher mindfulness may buffer the negative impact of early adverse experiences on psychological resilience.

This study aims to better understand the long-term consequences of these factors on the mental and emotional wellbeing of female university students by revealing the effects of ACEs on psychological resilience and mindfulness. In addition, examining the role of mindfulness in relation to psychological resilience will make a significant contribution, especially in developing psychological support and intervention strategies. In this context, this research aims to provide important data for enhancing the effectiveness of psychological support processes.

## Methods

### Participants and procedure

This study employed a combination of convenience sampling, purposeful sampling, and snowball sampling strategies to reach a target population of female university students aged 18–21 enrolled in a public vocational school in Bursa, Turkey. Participants were recruited through widely used social media platforms, including university forums, student WhatsApp groups, and Telegram channels. This approach was chosen due to post-pandemic constraints, where in-person recruitment was neither practical nor ethically advisable, and online platforms allowed for broad and rapid access to participants.

While this method facilitated the inclusion of a wide range of students, it also carries limitations. One possible limitation of the sampling method used in this study is the risk of sampling bias. Since participants were primarily recruited through online platforms, the sample may have leaned toward students who are more comfortable with digital tools and actively engaged in virtual communication. This situation suggests that students with limited internet access or those who struggle with digital tools may not have been adequately represented in the study. To reduce this possibility and obtain a more balanced sample, announcements were sent explicitly to student groups from different departments and at various grade levels.

A total of 330 students were invited to participate. Among them, 255 met the inclusion criteria and completed the online questionnaire fully, yielding a response rate of 77.3%. The study did not offer any incentives—financial or academic—ensuring that participation remained entirely voluntary and free from pressure.

Inclusion criteria: Female undergraduate students aged 18–21, currently enrolled in the vocational school, with no prior formal training in mindfulness or meditation, who provided informed consent via Google Forms.

Exclusion criteria: Male students, students younger than 18 or older than 21, individuals with prior mindfulness training, and those who declined or incompletely filled out the survey.

The majority of participants (81.6%) self-reported a middle socioeconomic status. The mean age of the sample was 20.99 years (SD = 5.03).

### Data collection procedure

Data were collected anonymously between May and September 2023 via Google Forms. The initial page of the online survey provided a clear outline of the study's objectives, ethical standards, and the voluntary nature of participation. Participants were required to give informed digital consent prior to accessing the questionnaire. To maintain data integrity, only one submission was permitted per IP address. Importantly, no personally identifiable information (such as names or ID numbers) was collected. Participants were reassured that their anonymous responses would not impact their academic status. The survey included standardized instruments measuring: ACEs-TR, Childhood Trauma Questionnaire (CTQ)-TR, Adolescent and Adult Mindfulness Scale (AAMS-TR), and The Resilience Scale for Adults (RSA-TR). The final sample size of *n* = 255 is sufficient for correlation, regression, and mediation analyses. According to a priori power analysis using G^*^Power (*f*^2^ = 0.05, α = 0.05, power = 0.80), this sample offers adequate statistical power. Effect sizes (Cohen's d and *R*^2^) were calculated and reported. Bootstrapping with 5,000 resamples was used in mediation analysis to obtain robust estimates and confidence intervals for indirect effects.

Utilizing snowball sampling through social media has proven to be an effective method for identifying participants. However, this approach may have introduced selection bias, as students who are more engaged in online communities or who possess greater digital literacy skills may be overrepresented in the sample. This limitation is noted in the discussion section, and it is important to exercise caution when generalizing the results to wider populations. Before analysis, missing data (<5%) were handled via listwise deletion. Outliers were assessed using *z*-scores and Mahalanobis distance, with no extreme values requiring removal. Assumptions of normality (skewness and kurtosis < ±1.5), multicollinearity (VIF < 2), and homoscedasticity (scatterplots of residuals) were also checked.

### Ethical considerations

The study protocol was approved by the University Institutional Ethics Committee (Approval No: E-50631952-044-2206). All research procedures were conducted in accordance with the Declaration of Helsinki.

### Data collection tools

#### Sociodemographic data form

A form was created to gather data on the age and socio-economic status of girl university students and their families.

#### Childhood Trauma Questionnaire (CTQ)-TR

It was developed by Bernstein et al. (1998) to measure childhood abuse and neglect experiences of individuals. Three items of the scale measure minimization. The scale consists of a total of 28 items using a five-point Likert type (1 = never; 5 = very often). It assesses the dimensions of physical abuse, emotional abuse, sexual abuse, physical neglect, and emotional neglect in childhood. The higher the score obtained from the scale, the greater the severity of the abuse and neglect experienced. The Turkish reliability and validity study of the scale was conducted by Sar et al. (2012). As a result of the reliability study, the Cronbach alpha coefficient calculated for internal consistency was determined to be 0.93. For test-retest reliability studies, the scale was re-administered to the participants at a 2-week interval, and it was found that the CTQ-TR demonstrated a high level of test-retest reliability (*r* = 0.90) (Sar et al., 2012). The correlation coefficients ranged from 0.90 for physical and emotional abuse, 0.85 for emotional neglect, 0.85 for sexual abuse, 0.75 for physical neglect, and 0.77 for emotional neglect (Sar et al., 2012). In the current study, the correlation coefficients ranged from 0.88 for physical and emotional abuse, 0.87 for emotional neglect, 0.92 for sexual abuse, and 0.70 for physical neglect.

#### The Resilience Scale for Adults (RSA-TR)

It was developed by Friborg et al. (2003), and Basim and Cetin (2011) conducted a Turkish validity and reliability study of the scale. The scale items were evaluated as described in the original study. To eliminate acquaintance bias, the five-point Likert scale opposite the answers can be used and evaluated as desired. To increase psychological resilience as scores increase, the answer boxes were evaluated from left to right using the scale of 1–5. The original scale had a total Cronbach alpha coefficient of 0.86. The current study aims to increase psychological resilience as the scores increase (Basim and Cetin, 2011). In the current study, the total Cronbach alpha value of RSA-TR was found to be 0.84.

#### ACEs Turkish form (ACEs-TR)

It was developed during the CDC-Kaiser Permanente ACEs study conducted between 1995 and 1997 (Felitti et al., 1998). In 2018, Gündüz et al. conducted a Turkish validity and reliability study on the Childhood Adverse Experiences Scale, which consists of 10 questions. The questions are answered with a “yes” or left blank if the answer is “no.” No cut-off value has been recommended for ACEs-TR. The Cronbach alpha value calculated to determine the internal consistency was reported as 0.74 (Gündüz et al., 2018). In the current study, the Cronbach alpha value was found to be 0.78.

Two factors primarily drove the decision to utilize both scales in conjunction. The objective was to facilitate a more comprehensive and multidimensional investigation into childhood traumas. Secondly, it was recognized that the features measured by the two scales differed significantly. To illustrate this point, the CTQ scale was developed to assess different dimensions of childhood traumas, including physical, emotional, and sexual abuse, as well as physical and emotional neglect. In summary, the CTQ scale provides a comprehensive and detailed insight into the specific types of abuse an individual has experienced during childhood. The ACEs-TR scale focuses on more general adverse experiences an individual may have faced during childhood. The ACEs scale provides data on a broader range of events beyond abuse, including physical or psychological bullying during childhood, domestic violence, and the loss of a parent.

### Adolescent and Adult Mindfulness Scale (AAMS-TR)

The scale, developed by Droutman et al. (2018), underwent validation and reliability testing for its Turkish adaptation by Arslan et al. (2020). This scale is designed to assess conscious awareness and comprises four key components: (1) attention and awareness, (2) non-reactivity, (3) non-judgment, and (4) self-acceptance.

In the original scale, there are four dimensions: the first dimension comprises nine items, the second dimension comprises three items, the third dimension comprises four items, and the fourth dimension comprises three items. The scale employs a five-point Likert-type rating system (1 = never true, 5 = always true). Higher scores on all subscales indicate a greater presence of the measured attribute (Arslan et al., 2020).

The results of the confirmatory factor analysis confirmed the validity of the four-factor structure of the Turkish version of the scale, which includes 18 items. In this version, Item 1 was omitted. The internal consistency coefficient values of the Cronbach alpha for the scale ranged from 0.64 to 0.84, while the construct reliability values ranged from 0.80 to 0.92. The Turkish version of the AAMS-TR comprises 18 items divided into four sub-dimensions (Arslan et al., 2020). In this study, the Cronbach alpha values of AAMS-TR are as follows: The Cronbach alpha value for attention and awareness is 0.83, for being non-reactive is 0.79, for being non-judgmental is 0.84, for self-acceptance is 0.74, and for minimization is 0.50.

### Data analysis

Descriptive statistics are presented as the mean and standard deviation (range: minimum to maximum) for quantitative data and as frequency and percentage for qualitative data. Relationships between variables were assessed using the Pearson correlation coefficient. Stepwise multiple linear regression analysis was conducted to investigate the variables influencing the RSA and AAMS scales. The dependent variable is the psychological resilience of female students, and the independent variables are mindfulness levels (AAMS), ACEs, and childhood traumas (CTQ). The significance level was set at α = 0.05. The data were subjected to a series of statistical procedures. Firstly, a correlation test was performed, adopting a significance level of 0.05. Secondly, a multivariate linear regression analysis was conducted. Statistical analysis of the data was carried out using the IBM SPSS 28.0 statistical software package (IBM Corp., Released 2021. IBM SPSS Statistics for Windows, version 28.0. Armonk, NY: IBM Corp.). Additionally, a mediation analysis was conducted using the PROCESS macro (version 4.2) (Model 1) in IBM SPSS 29.0.2.0 (IBM Corp. Released 2023. IBM SPSS Statistics for Windows, version 29.0.2.0 Armonk, NY: IBM Corp.) to evaluate whether the effect of the independent variables (CTQ_minimization, CTQ_emotional abuse, CTQ_physical abuse, CTQ_physical neglect, CTQ_emotional abuse, CTQ_sexual abuse, age) on the dependent variable (RSA_total) was transmitted through a mediator variable (AAMS_attention and awareness, AAMS_being non-reactive, AAMS_being non-judgmental, AAMS_self-accepting).

## Results

### Descriptive data

Table 1 presents the descriptive statistics of the variables, including the mean scores of the scales and their standard deviations.

**Table 1:** Descriptive statistics of the scale scores.

**Variables**	**Mean**	**Standard deviation (SD)**	**Min**.	**Max**.
^*^ACEs_total	1.68	2.06	0.00	9.00
^**^RSA_perception of the self	21.90	3.45	11.00	30.00
RSA_planned future	13.91	2.67	7.00	20.00
RSA_structured style	13.95	2.32	8.00	19.00
RSA_social competence	22.16	3.79	13.00	30.00
RSA_family cohesion	20.42	4.30	6.00	30.00
RSA_social resources	26.88	4.06	13.00	35.00
RSA_total	119.22	13.47	92.00	157.00
^***^AAMS_attention and awareness	33.21	5.02	17.00	40.00
AAMS_being non-reactive	10.43	2.66	3.00	15.00
AAMS_being non-judgmental	15.35	2.89	8.00	20.00
AAMS_self-accepting	9.67	2.65	3.00	15.00
^****^CTQ_minimization	0.62	0.84	0.00	3.00
CTQ_emotional abuse	7.81	4.03	5.00	25.00
CTQ_physical abuse	6.06	2.86	5.00	20.00
CTQ_physical neglect	6.80	2.58	5.00	16.00
CTQ _ emotional neglect	10.93	4.61	5.00	24.00
CTQ_sexual abuse	6.20	3.21	5.00	25.00

* Adverse childhood experiences;

** Resilience Scale for Adults;

*** Adolescent and Adult Mindfulness Scale;

**** Childhood Trauma Questionnaire.

The mean score for ACEs was 1.68, and for psychological resilience, it was 119.22. When examining the mindfulness sub-dimensions, the “attention and awareness” sub-dimension had the highest value, with an average score of 33.21, while the “self-acceptance” sub-dimension had the lowest average, at 9.67. Among the mean scores for childhood trauma experiences, emotional neglect (10.93) had the highest score, followed by emotional abuse (7.81).

The overall mean score for ACEs indicates that the female students in the study reported low levels of adverse experiences (1.68). The examination of the types of childhood trauma revealed that participants experienced emotional neglect most frequently, followed by emotional abuse. In addition, the participants demonstrated a high average level of psychological resilience (119.22).

Table 2 provides details of the correlation between the ACEs scale total and other scale scores.

**Table 2:** Correlation table between ACEs total scale and other scale scores.

**Variables**			**ACEs total**	**CTQ**					
				**Minimization**	**Emotional abuse**	**Physical abuse**	**Physical neglect**	**Emotional neglect**	**Sexual abuse**
ACEs total		*r*	–	−0.402^*^	0.610	0.416^*^	0.464^*^	0.638^*^	0.487^*^
		*p*	–	<0.001	<0.001	<0.001	<0.001	<0.001	<0.001
RSA	Perception of the self	*r*	−0.002	0.231^*^	−0.133^**^	−0.043	−0.058	−0.151^**^	−0.067
		*p*	0.972	<0.001	0.033	0.499	0.360	0.015	0.283
	Planned future	*r*	−0.190^*^	0.302^***^	−0.189^**^	−0.147^**^	−0.180^*^	−0.291^*^	−0.171^**^
		*p*	0.002	<0.001	0.002	0.019	0.004	<0.001	0.006
	Structured style	*r*	−0.140^**^	0.187^**^	−0.119^***^	−0.165^*^	−0.216^*^	−0.176^*^	−0.103
		*p*	0.026	0.003	0.059	0.008	0.001	0.005	0.101
	Social competence	*r*	0.006	0.174^*^	−0.103	−0.045	−0.162^*^	−0.190^*^	−0.120^***^
		*p*	0.927	0.005	0.101	0.471	0.010	0.002	0.056
	Family cohesion	*r*	−0.576^*^	0.453^*^	−0.547^*^	−0.407^*^	−0.513	−0.704^*^	−0.358^*^
		*p*	<0.001	<0.001	<0.001	<0.001	<0.001	<0.001	<0.001
	Social resources	*r*	−0.440^*^	0.370^*^	−0.444^*^	−0.428^*^	−0.467^*^	−0.568^*^	−0.411^*^
		*p*	<0.001	<0.001	<0.001	<0.001	<0.001	<0.001	<0.001
	Total	*r*	−0.377^*^	0.456^*^	−0.429^*^	−0.340^*^	−0.437^*^	−0.576^*^	−0.341^*^
		*p*	<0.001	<0.001	<0.001	<0.001	<0.001	<0.001	<0.001
AAMS	Attention and awareness	*r*	0.002	0.188^*^	−0.018	−0.137^*^	−0.340^*^	−0.177^*^	−0.178^*^
		*p*	0.971	0.003	0.781	0.029	<0.001	0.005	0.004
	Being non-reactive	*r*	0.091	0.109^***^	−0.032	−0.157^**^	−0.123^**^	0.068	−0.042
		*p*	0.149	0.083	0.607	0.012	0.049	0.277	0.508
	Being non-judgmental	*r*	−0.017	0.184^*^	−0.018	−0.088	−0.216^*^	−0.112^***^	−0.069
		*p*	0.782	0.003	0.779	0.161	0.001	0.075	0.275
	Self-accepting	*r*	0.132^**^	−0.131^**^	0.137^**^	−0.005	0.031	0.144^**^	0.065
		*p*	0.036	0.036	0.029	0.943	0.625	0.022	0.302

* < 0.01;

** < 0.05;

*** < 0.1.

ACEs, adverse childhood experiences; RSA, Resilience Scale for Adults; AAMS, Adolescent and Adult Mindfulness Scale; CTQ, Childhood Trauma Questionnaire.

A weak to moderate inverse relationship was found between the ACEs total score and both the total score of the RSA and its sub-dimensions. A positive, weakly significant relationship was observed between the ACEs total score and only the self-accepting score (*r* = 0.132; *p* = 0.036) among the sub-dimensions of the AAMS. There was a moderate correlation between the CTQ minimization subscale score and RSA family cohesion (*r* = 0.453; *p* < 0.001) and total score (*r* = 0.456; *p* < 0.001).

The CTQ emotional abuse subscale score showed a moderate, significant inverse relationship with RSA family cohesion, social resources, and total scores (*r* = −0.547; *p* < 0.001; *r* = −0.444; *p* < 0.001; *r* = −0.429; *p* < 0.001, respectively). While the CTQ emotional abuse subscale score was weakly and positively correlated with the AAMS self-accepting subscale (*r* = 0.137; *p* = 0.029), no significant relationship was found with other subscales.

A moderate significant inverse correlation was observed between the CTQ physical abuse subscale score and the RSA social resources score (*r* = −0.428; *p* < 0.001). At a very weak level, the CTQ physical abuse subscale score was inversely related to the AAMS attention and awareness (*r* = −0.137; *p* = 0.029) and being non-reactive (*r* = −0.157; *p* = 0.012) subscales, with no significant correlation found with other subscales.

There was a moderate, inverse association observed between the CTQ-physical neglect subscale and the scores of RSA-family cohesion (*r* = −0.513; *p* < 0.001), social resources (*r* = −0.467; *p* < 0.001), and RSA total (*r* = −0.437; *p* < 0.001). While an inverse relationship existed between the CTQ-physical neglect subscale score and the AAMS-attention and awareness (*r* = −0.340; *p* < 0.001), as well as being non-reactive (*r* = −0.123; *p* = 0.049) and being non-judgmental (*r* = −0.216; *p* = 0.001) subscales, there was no significant relationship with the self-accepting subscale.

The CTQ emotional neglect subscale score showed a noteworthy inverse correlation with RSA-family cohesion (*r* = −0.704; *p* < 0.001) and established a moderate significant inverse association with the social resources score (*r* = −0.568; *p* < 0.001) and RSA total (*r* = −0.576; *p* < 0.001). A negative correlation was observed between the CTQ-emotional neglect subscale score and AAMS-attention and awareness (*r* = −0.177; *p* = 0.005). Conversely, a positive correlation was found with the self-accepting subscale (*r* = 0.144; *p* = 0.022). No significant correlation was identified with the other subscales.

There was a moderate significant inverse relationship between the CTQ sexual abuse subscale score and the RSA social resources score (*r* = −0.411; *p* < 0.001). Additionally, a very weak significant inverse relationship existed between the CTQ emotional abuse subscale score and the AAMS attention subscale (*r* = −0.178; *p* = 0.004). No significant relationship was found with the other subscales.

Table 3 presents the results of the multiple linear regression analyses examining the associations between the predictor variables and the total score and sub-dimensions of RSA.

**Table 3:** Multiple linear regression analysis results of variables related to RSA total and sub-dimensions.

**Variables**	**Unstandardized beta**	**Std. error**	**95.0% confidence interval for beta**		**Standardized beta**	***t***	***P*-value**
			**Lower bound**	**Upper bound**			
**Dependent** = **RSA_ perception of the self Adj** *R*^2^ = **0.125;** *F*_(4, 249)_ = **10.201;** ***p***<**0.001**							
Constant	15.704	1.711	12.334	19.075	–	9.176	<0.001^*^
AAMS attention and awareness	0.179	0.043	0.096^***^	0.263	0.261	4.214	<0.001^*^
AAMS self-accepting	−0.199	0.080	−0.357	−0.041	−0.153^*^	−2.481	0.014^**^
CTQ _ minimization	0.568	0.253	0.071	1.065	0.139^*^	2.249	0.025^**^
Age	0.086	0.041	0.004	0.167	0.125^*^	2.075	0.039^**^
**Model dependent** = **RSA_ planned future Adj** *R*^2^ = **0.132;** *F*_(3, 250)_ = **13.771;** ***p***<**0.001**							
Constant	11.427	1.226	9.012	13.842	–	9.320	<0.001^*^
CTQ _ minimization	0.572	0.232	0.116	1.028	0.181	2.471	0.014^**^
AAMS attention and awareness	0.094	0.032	0.031	0.156	0.176	2.936	0.004^*^
CTQ _ emotional neglect	−0.089	0.042	−0.172	−0.006	−0.154	−2.108	0.036^**^
**Model dependent** = **RSA_ structured style Adj** *R*^2^ = **0.190;** *F*_(5, 248)_ = **12.833;** ***p***<**0.001**							
Constant	7.558	1.125	5.342	9.775	–	6.716	<0.001^*^
AAMS attention and awareness	0.143	0.029	0.087	0.199	0.309	5.011	<0.001^*^
AAMS self-accepting	−0.128	0.052	−0.232	−0.025	−0.147	−2.446	0.015^**^
AAMS being non-reactive	0.181	0.054	0.073	0.288	0.207	3.316	0.001^*^
ACE_total	−0.167	0.065	−0.295	−0.040	−0.149	−2.584	0.010^*^
Age	0.061	0.027	0.008	0.113	0.131	2.282	0.023^**^
**Model dependent** = **RSA_ social competence Adj** *R*^2^ = **0.192;** *F*_(4, 249)_ = **15.983;** ***p***<**0.001**							
Constant	10.267	1.989	6.350	14.185	–	5.162	<0.001^*^
AAMS attention and awareness	0.160	0.048	0.065	0.255	0.211	3.310	0.001^*^
AAMS being non-judgemental	0.297	0.083	0.134	0.460	0.227	3.588	<0.001^*^
age	0.146	0.043	0.062	0.230	0.193	3.411	0.001^*^
CTQ _ emotional neglect	−0.094	0.047	−0.187	0.000	−0.114	−1.978	0.049^**^
**Model dependent** = **RSA_ family cohesion Adj** *R*^2^ = **0.528;** *F*_(3, 250)_ = **95.316;** ***p***<**0.001**							
Constant	27.717	0.601	26.533	28.901	–	46.109	<0.001^*^
CTQ _ emotional neglect	−0.475^*^	0.058	−0.589	−0.361	−0.511	−8.221	<0.001^*^
ACE_total	−0.400^*^	0.118	−0.633	−0.167	−0.192	−3.383	0.001^*^
CTQ physical neglect	−0.209^*^	0.092	−0.390	−0.027	−0.122	−2.264	0.024^**^
**Model dependent** = **RSA_ social resources Adj** *R*^2^ = **0.420;** *F*_(5, 248)_ = **37.644;** ***p***<**0.001**							
Constant	27.882	1.571	24.787	30.977	–	17.743	<0.001^*^
CTQ emotional neglect	−0.384	0.049	−0.480	−0.287	−0.436	−7.820	<0.001^*^
CTQ sexual abuse	−0.218	0.071	−0.357	−0.079	−0.170	−3.082	0.002^*^
AAMS attention and awareness	0.124	0.041	0.043	0.204	0.153	3.020	0.003^**^
CTQ physical abuse	−0.204	0.085	−0.372	−0.037	−0.138	−2.399	0.017^**^
AAMS self-accepting	0.172	0.077	0.021	0.324	0.113	2.240	0.026^**^
**Model dependent** = **RSA_total Adj** *R*^2^ = **0.442;** *F*_(5, 248)_ = **41.114;** ***p***<**0.001**							
Constant	101.385	6.006	89.554	113.215	–	16.879	<0.001^*^
CTQ emotional neglect	−1.381	0.148	−1.673	−1.089	−0.473	−9.316	<0.001^*^
AAMS attention and awareness	0.577	0.143	0.295	0.859	0.215	4.030	<0.001^*^
Age	0.362	0.126	0.113	0.610	0.135	2.866	0.005^*^
AAMS being non-judgemental	0.606	0.244	0.125	1.086	0.130	2.480	0.014^*^
CTQ sexual abuse	−0.502	0.215	−0.925	−0.079	−0.118	−2.336	0.020^*^
**Model summary**							
Std. error	3.23	2.49	2.09	3.41	2.95	3.09	
*R*	0.372	0.377	0.453	0.452	0.730	0.657	
*R* ^2^	0.139	0.142	0.206	0.204	0.534	0.431	
Adj *R*^2^	0.125	0.132	0.190	0.192	0.528	0.420	
*F*	10.021	13.771	12.833	15.983	95.316	37.644^*^	
p	<0.001	<0.001	<0.001	<0.001	<0.001	<0.001	

b, unstandardized regression coefficients; beta, standardized regression coefficients.

ACEs, Adverse Childhood Experience; RSA, the Resilience Scale for Adults; AAMS, Adolescent and Adult Mindfulness Scale; CTQ, Childhood Trauma Questionnaire.

* < 0.01;

** < 0.05;

*** < 0.1.

The perception of the self sub-dimension of RSA was significantly predicted by the CTQ minimization subscale and age. The planned future sub-dimension was significantly influenced by both CTQ minimization and emotional neglect, with minimization showing a stronger effect [*F*_(3, 250)_ = 13.771, *p* < 0.001]. The structured style sub-dimension was significantly associated with CTQ physical neglect and age [*F*_(5, 248)_ = 12.833, *p* < 0.001]. For social competence, only CTQ emotional neglect was a significant predictor, and its contribution to the model exceeded that of the total ACEs score [*F*_(4, 249)_ = 15.983, *p* < 0.001]. In predicting the family cohesion sub-dimension, significant predictors included the ACEs total score, CTQ physical neglect, and emotional neglect, with emotional neglect having the strongest impact, and physical neglect the weakest [*F*_(3, 250)_ = 95.316, *p* < 0.001]. Finally, the total RSA score was significantly associated with the CTQ sub-dimensions minimization, emotional neglect, and sexual abuse, with emotional neglect contributing the most to the model.

The findings indicated that the RSA subdimension “perception of the self” was significantly predicted by the AAMS subdimensions “attention and awareness” and “self-accepting,” as well as by age, with “attention and awareness” emerging as the most influential predictor among these. The RSA sub-dimension “social competence” was significantly predicted by the AAMS sub-dimensions “attention and awareness” and “being non-judgmental,” with “being non-judgmental” having the highest relative weight in the model. When examining predictors of the RSA total score, significant associations were observed with the AAMS sub-dimensions “attention and awareness,” “being non-judgmental,” and age. Among the significant predictors in all models, “self-accepting” was consistently found to have the lowest standardized beta coefficient.

In this study, the role of moderator variables, specifically the sub-dimensions of the mindfulness scale, in the relationship between the independent variables CTQ_minimization, CTQ_emotional abuse, CTQ_physical abuse, CTQ_physical neglect, emotional neglect, CTQ_sexual abuse, and the dependent variable RSA_total was examined. The analysis results show that the moderator effects were statistically significant. Firstly, when the moderator effect of mindfulness_being non-reactive in the relationship between CTQ_emotional abuse and RSA_total was examined, the interaction term was significant (β = −0.138, *p* = 0.035). As the value of mindfulness_being non-reactive increases, the negative effect of CTQ_emotional abuse on RSA_total becomes stronger. In the relationship between CTQ_physical neglect and RSA_total, both mindfulness_attention and awareness (β = −0.135, *p* = 0.006) and mindfulness_being non-reactive (β = −0.247, *p* = 0.018) showed significant moderator effects. As the of mindfulness_attention and awareness and mindfulness_being non-reactive increase, the effect of CTQ_physical neglect on RSA_total becomes more negative. These findings reveal that the moderator variables (attention-awareness, being non-reactive) significantly shape the relationships between the independent variables (CTQ_emotional abuse, CTQ_physical abuse, CTQ_physical neglect) and the dependent variable (RSA _total).

## Conclusion and discussion

The study also examined the effects of childhood traumas and ACEs on the psychological resilience of female university students. The findings revealed that the psychological resilience of these students was significantly affected by childhood traumas. When the relationship between childhood traumas and psychological resilience was examined, a negative association was found between emotional neglect, emotional abuse, and physical neglect (*r* = −0.576; *p* < 0.001; *r* = −0.429; *p* < 0.001; *r* = −0.437; *p* < 0.001, respectively). Psychological resilience is the ability to return to a normal state after facing difficulties. However, childhood traumas can weaken this resilience by increasing automatic negative thoughts in individuals (Yu et al., 2022; Chang et al., 2021; Morgan et al., 2021). Children who experience ACEs, including abuse and neglect, tend to have lower resilience. This is because ACEs and childhood traumas negatively impact the ability to manage stress and regulate emotions, which are important skills related to psychological resilience (Barnová et al., 2019). As a result, female university students who experience ACEs and childhood traumas are likely to have low psychological resilience. Related research has shown that maltreatment, abuse, and neglect negatively impact the resilience of many adolescents and adults (Chen et al., 2021b; Costa et al., 2013; Li C. et al., 2023).

In the current study, when the effects of childhood traumas on psychological resilience were examined, it was found that the emotional neglect dimension had the highest effect [*F*_(4, 249)_ = 15.983, *p* < 0.001], while physical neglect had the lowest impact. These findings are consistent with the existing literature. In their study on university students in China, Liang et al. (2018a,b) found that individuals who experienced emotional neglect from their parents during childhood had significantly lower levels of hope, optimism, and self-efficacy. Similarly, Dimitriu et al. (2023) reported that ACEs negatively affected psychological resilience in adolescents, with the most significant effect being domestic violence. Children who were abused and neglected were more likely to have neurological, psychological, and cognitive disorders compared to their non-abused peers (Child Welfare Information Gateway, 2010). Children exposed to ACEs may exhibit increased levels of stress and anxiety due to altered neurobiological responses, particularly dysregulation of the hypothalamic-pituitary-adrenal (HPA) axis, which is responsible for the stress response (Gunnar and Quevedo, 2007). Chronic activation of the stress response may lead to increased sensitivity to stressors, interfering with emotional regulation and coping strategies (Ursu and Măirean, 2022; Sun et al., 2023). Experiences of abuse and neglect in childhood are particularly damaging and can be irreversible; however, the resilience of children and adolescents can be supported and enhanced (Kairyte et al., 2023). According to Phillips et al. (2019), resilience is strengthened by external protective factors such as family, school, and peer groups. The adverse effects of ACEs can be reduced or prevented by developing social and emotional skills that support psychological resilience in children and adolescents (Chang et al., 2021). As a result, the negative effects of childhood trauma or ACEs on psychological resilience may be more pronounced, especially for female university students. In future interventions, it is recommended that personalized support programs be developed to increase psychological resilience by considering individuals‘ ACEs history. Such interventions may support students' academic and personal success and improve their long-term psychological wellbeing.

The study examined the relationship between ACEs and childhood traumas of female university students and their levels of mindfulness. It was found that the type of childhood trauma that most affected the mindfulness of female students was physical neglect (*r* = −0.340). The relationship was weak but negative with other childhood traumas. Notably, in the mindfulness dimensions, attention and awareness were the sub-dimensions most affected by traumatic experiences and physical neglect (*r* = −0.340). The findings are consistent with previous studies showing that childhood traumas and adverse experiences harm the mindfulness of adolescents and adults (Yildiz and Demir, 2022; Fitzgerald, 2022). For example, Çelik (2020) identified a significant negative correlation between physical abuse and mindfulness. Attention and awareness are cognitive abilities that encompass the capacity to focus, sustain attention, and be aware of one's surroundings, thoughts, feelings, and emotions. This finding suggests that girls who experienced physical neglect during childhood may have difficulties with concentration, attention span, and awareness of their surroundings, thoughts, feelings, and emotions. This study is consistent with other studies that found a negative relationship between types of abuse and neglect and mindfulness (Huang C. et al., 2021; Frewen et al., 2012; McKeen et al., 2023; Fox et al., 2024). These findings support the idea that childhood traumas generally negatively affect mindfulness. Individuals who experience trauma may become hypersensitive to potential danger or behave dissociatively, which decreases their ability to maintain attention and awareness (Ely et al., 2023; Voith et al., 2020). The study found a significant and negative relationship between female university students' “non-judgmental” levels and physical neglect (*r* = −0.216). This finding indicates that individuals who have experienced physical neglect have difficulty avoiding judgment, an important component of conscious awareness. Being non-judgmental means accepting one's thoughts, feelings, and experiences at the moment while avoiding the tendency to evaluate or criticize. However, it is evident that female university students who have experienced physical neglect have difficulties in achieving this type of awareness and tend to judge their inner experiences more. This suggests that physical neglect may negatively affect the development of conscious awareness skills. This finding is similar to the results of the study by Ünal (2021). The literature indicates a relationship between non-judgmentalism and self-compassion. Self-compassion relates to the non-judgmental aspect of mindfulness. According to Neff (2003a), self-compassion consists of three basic elements: self-kindness, common humanity, and awareness. It involves treating oneself with care and understanding without self-criticism during difficult times. Additionally, acknowledging challenging experiences as an inherent part of life and focusing on constructive problem-solving rather than dwelling on distress may support psychological adaptation (Neff, 2003a,b). Similarly, Boughner et al. (2016) found negative associations between sexual abuse and mindfulness as well as non-judgmentality. Higher levels of trauma exposure throughout the lifespan were associated with lower levels of “mindfulness and non-judgmentality,” consistent with the findings of Roche et al. (2019). The study determined a negative association between childhood trauma and observation, mindfulness, and non-judgmentality.

In the study, some traumatic experiences—emotional neglect, emotional abuse, and the ACEs total score—were surprisingly positively associated with self-acceptance (*r* = +0.144; *r* = +0.137; *r* = +0.132, respectively). This result is unexpected. Related studies in the literature provide opposing evidence. For example, Barros et al. (2022) concluded that emotional neglect and abuse experienced by university students during childhood harmed the “self-acceptance” aspect of their psychological wellbeing. However, some studies support the current findings. Other studies in the literature suggest that traumatic events experienced in childhood may have a positive effect on post-traumatic growth. Post-traumatic growth is characterized by a better level of functioning and development in certain areas of life after a traumatic event. Research has shown that people can be affected by trauma in different ways. Post-traumatic events can prompt individuals to re-evaluate their lives, relationships, and sense of self. They can also lead to positive psychological change and post-traumatic growth (Tedeschi and Calhoun, 2004; Westphal and Bonanno, 2007). Research suggests that individuals with trauma histories may cognitively process adverse experiences without a negative appraisal, potentially as a coping strategy to reduce psychological distress, though De Moraes et al. (2023) note that this mechanism may reflect maladaptive avoidance.

Gruhn and Compas (2020) demonstrate in their meta-analysis that childhood maltreatment is generally associated with poor emotion regulation, increased avoidance, emotional suppression, and the expression of negative emotions in response to stress.

In this study, the mediating role of mindfulness in the effect of ACEs on psychological resilience was examined. The findings make a significant contribution to the clinical psychology and trauma literature by shedding light on the moderating role of mindfulness sub-dimensions in the effect of childhood traumatic experiences on psychological resilience. An unexpected finding was that the negative relationship between emotional abuse and psychological resilience strengthened as the level of being non-reactive increased (β = −0.138, *p* = 0.035). Being non-reactive describes the ability not to suppress thoughts and emotions but to accept and acknowledge them. In other words, among the aspects of mindfulness is the ability to experience thoughts and emotions without reactive responses. While it is generally expected that the “being non-reactive” dimension of mindfulness would serve as a protective factor, this result suggests that excessive emotional inhibition may amplify the effects of trauma. The “emotional suppression” hypothesis can explain this unexpected finding. Being non-reactive can be interpreted as emotional unresponsiveness; this pattern may reflect emotion regulation difficulties associated with traumatic stress responses, as discussed by Hayes and Feldman (2004). Boughner et al. (2016) noted that mindfulness non-reactivity was a significant mediator of the relationship between increased exposure to childhood trauma and heightened PTSD symptoms. From this perspective, it is believed that it may negatively affect psychological resilience by increasing post-traumatic stress disorder. The analyses revealed that the sub-dimensions of mindfulness significantly moderated the relationship between childhood physical neglect and psychological resilience. Specifically, female university students who scored higher on the sub-scales of attention and awareness (β = −0.135, *p* = 0.006) and being non-reactive (β = −0.247, *p* = 0.018) experienced more substantial adverse effects on resilience as measured by the physical neglect sub-scale of the CTQ, which is an important finding that can be described as the “paradoxical trauma effect” in the mindfulness literature. Mindfulness-induced hyperawareness of trauma memories reflects the complexities involved in processing traumatic experiences. While the goal of mindfulness practices is often to improve emotional regulation and promote psychological healing, such approaches can paradoxically lead to an increase in the frequency and intensity of intrusive memories associated with past traumas. This paradox is highlighted by research showing that mindfulness-based stress reduction (MBSR) programs can improve symptoms of post-traumatic stress disorder (PTSD). However, they may also initially increase the activation of traumatic memories, resulting in heightened distress among participants (Kearney et al., 2012; Gallegos et al., 2020). Research has shown that veterans who participate in mindfulness programs often report more intense confrontations with repressed trauma, illustrating a complex dynamic in therapeutic settings (Kearney et al., 2012). This counterintuitive pattern, which we conceptualize as mindfulness-exacerbated trauma vulnerability, aligns with emerging evidence of meditation-induced distress in trauma-exposed populations (Lindahl et al., 2017) and challenges the assumption of universal efficacy for mindfulness interventions (Van Dam et al., 2018). Various studies have also identified the potential adverse effects of mindfulness practices (Shapiro, 1992; Lindahl et al., 2017). This result contradicts the classical view that mindfulness is a protective factor in general (Kabat-Zinn, 2003). Wong et al. (2018) conducted a systematic review that found mindfulness is not a “completely risk-free” practice. It should be used with caution, especially for those with psychiatric disorders, those with neurological predispositions (e.g., epilepsy), and those with a history of trauma. In particular, the finding that excessive introspective mindfulness may increase rumination among individuals with histories of childhood physical neglect supports the ‘negative side' hypothesis. Mindfulness is generally beneficial, but under certain circumstances, for specific individuals or at certain levels, it may be harmful, have costs, or have undesirable effects (Britton, 2019).

Current research findings suggest that sub-dimensions of mindfulness have unexpectedly negative moderating effects on the relationship between childhood traumatic experiences and resilience. These results open a new discussion that questions the “all-improving” assumption in the mindfulness literature and supports the concept of “disadaptive mindfulness.” Mindfulness is not a universal healer—it may trigger disadaptive processes in the context of trauma. It is important to approach this conclusion with caution, as the study's methodology may have influenced the degree of traumatic experience and the scales used. There may also be differences in trauma severity among the participants included in the study, indicating the need to group participants according to trauma severity (e.g., CTQ severity scores). Examining threshold effects by comparing clinical and non-clinical groups may also be necessary. Mindfulness components such as “non-reactivity” may function differently in collectivist cultures where emotional suppression is the norm. The findings can be replicated in other Western countries using the same methodology and can be re-examined with different methodologies and measurement tools.

## Limitations and recommendations

This study has generalizability and self-report bias limitations that should be considered when interpreting the findings. One key limitation is the use of a social media-based snowball sampling method, which may have introduced selection bias. Since participants were recruited from similar social and educational environments through online networks, the sample may not represent the broader population of university students. As a result, the generalizability of the findings to different demographic or cultural contexts is limited. This limitation should be taken into account when interpreting the results. In particular, focusing only on female students may overlook the effects of gender. In addition, conducting the study at only one vocational school limits the understanding of how results may vary across different types of universities or institutions. Future research should include a more diverse group of participants, such as males, graduate students, non-university youth, and individuals from various educational institutions. This broader approach could enhance the generalizability of the findings and offer a more comprehensive perspective. Given these limitations, it is crucial to interpret the study's results with caution. Cultural, socioeconomic, and institutional differences among universities and regions may influence psychological resilience and related factors. Future research should include more diverse demographic groups (e.g., different age ranges, genders, education levels, and socioeconomic backgrounds) and multi-center sampling to increase external validity. Furthermore, cross-cultural studies will help determine whether the observed patterns are valid in different social contexts. Due to the cross-sectional methodology of the research, causal links between variables cannot be identified, making longitudinal studies necessary. Self-report data collection methods have well-known limitations, such as participants' biases, difficulty recalling the past, and inaccuracies in reporting information. Self-report measures may exhibit various biases that could affect the reported results, and some of these may be difficult to measure or control. Reliance on self-reported data introduces potential biases such as social desirability bias, recall bias, and subjective interpretation of survey items. Participants may under- or over-report specific experiences due to personal perceptions or stigma. Furthermore, resilience is a complex construct influenced by developmental and situational factors and may not be fully captured by a single self-report measure. To alleviate these limitations, future studies could include multi-method assessments (e.g., behavioral observations, clinician-rated scales, or peer reports) to complement self-reports, as well as longitudinal designs to track psychological resilience development over time and reduce immediate response biases. Objective measures (e.g., physiological markers of stress response) should be used where applicable. Furthermore, only one instrument was used in this study to assess the psychological resilience of female university students. Psychological resilience develops through a process influenced by age, gender, and type of trauma. More comprehensive research should be conducted to accurately determine the resilience levels of individuals who have experienced adverse childhood events. Future research should address the factors affecting psychological and trauma-focused psychological resilience in light of the individual's current situation (Klika and Herrenkohl, 2013). Therefore, to measure resilience in children exposed to various traumas (such as abuse, violence, maltreatment, or disasters), it is crucial to design an assessment tool that considers both the absence of trauma impact and the presence of trauma-focused protective factors. Careful consideration in the instrument's design must ensure objectivity, understandability, a conventional structure, transparent and objective language, adherence to format, formal recording, logical progression, and grammatical correctness. Psychological resilience tools commonly used in research are often inadequate for measuring true resilience factors in people with a history of trauma. Such instruments have primarily been validated on children and adolescents or young adults without traumatic experiences. Satapathy et al. (2022) suggest developing new measurement tools for psychological resilience appropriate for people with adverse experiences. In addition, it is recommended that more than one measurement tool be used in future studies. While these limitations do not invalidate the findings, they emphasize the need for cautious interpretation and further validation in various settings. Future research should aim to replicate these results in more heterogeneous samples and use mixed-method approaches to strengthen reliability and generalizability.

## Data Availability

The raw data supporting the conclusions of this article will be made available by the authors, without undue reservation.
